# Design Method to Structure Orthosis Design: Camptocormia Postural Brace Case Study

**DOI:** 10.1155/2019/3513947

**Published:** 2019-02-03

**Authors:** Ricardo Duarte, Jean-Pierre Nadeau, Antonio Ramos, Michel Mesnard

**Affiliations:** ^1^Université de Bordeaux, I2M, Esplanade des Arts et Métiers, 33405 Talence, France; ^2^Arts et Métiers, I2M, Esplanade des Arts et Métiers, 33405 Talence, France; ^3^Universidade de Aveiro, Campus Universitário de Santiago, 3810 Aveiro, Portugal

## Abstract

The orthosis is considered a class 1 medical device which often originates from a nonstructured development process. As these devices are mainly developed by small- and medium-sized enterprises, with no standard research method, the result can be an unadapted device which may not respond to the user's needs and which in the short term may be abandoned. One way to solve this problem is to define and apply standard rules and procedures throughout the development/design process. Although methodologies may solve the “empiricism” in orthosis design problems, these design strategies are not applied during orthosis development due to the particularities of this field and the difficulties in linking the required knowledge and the actors that may be present during the orthosis development. The objective of this work is to develop a methodology to structure the orthosis design process that takes into account both the device life cycle and the different stakeholders involved in the design process. A case study was used to validate the proposed methodology. It was applied to the development of an orthosis to treat a specific postural disorder called camptocormia, also known as bent spine syndrome. This disorder is characterized by the anteroflexion of the trunk and especially affects elderly people. Contrary to scoliosis, the characteristics of camptocormia are not permanent, which means that the patient is able to straighten his posture. A postural brace is used to treat this disorder which enables the patient to redress and maintain the correct upright posture of the trunk.

## 1. Introduction

The design of orthopaedic devices, or orthoses, may include particular requirements and design specifications, mainly related to the patients' varying characteristics, such as morphological changes or treatment evolution. The reason is that the orthosis is in permanent direct contact with the patient's body and consequently should be adaptable and comfortable.

Since orthoses are considered as a class 1 medical device, there are no strict rules and stages that should be respected during the development process [1, 2]. Consequently, orthosis development depends mainly on the “empirical knowledge” of the companies, which are mainly small- and medium-sized enterprises (SMEs). Additionally, the device may be poorly adapted to the user needs [3, 4]. One way to counteract this empiricism is to apply a design method that can guide the designer throughout the entire development process. However, due to the placing of the orthosis in the medical devices classification, there is no dedicated method for orthosis design.

One of the current challenges in the field of orthoses is to develop a device to treat camptocormia [5, 6]. Also known as bent spine syndrome, this pathology is characterized by the anteroflexion of the trunk in elderly populations. Camptocormia precludes the patients from having a normal daily life since the curved posture causes contraction of the diaphragm (fatigue problems) [5], reduces walking gait, results in a lack of social visual contact, etc.

The purpose of this study was to develop a design methodology devoted specifically to the orthosis and its application during the development of a brace to treat camptocormia.

## 2. Materials and Methods

Using the breakdown of the design process proposed by Pahl and Beitz [7, 8] as a base (task clarification, conceptual design, embodiment design, and detailed design), it was possible to develop a new design methodology for the development of the orthosis.

The first stage of the proposed methodology corresponds to task clarification, which may include the *definition of the orthosis stakeholder*, *the orthosis typology*, and *the orthosis life situations*.

### 2.1. Orthosis Development Stakeholder

**Figure 1:**
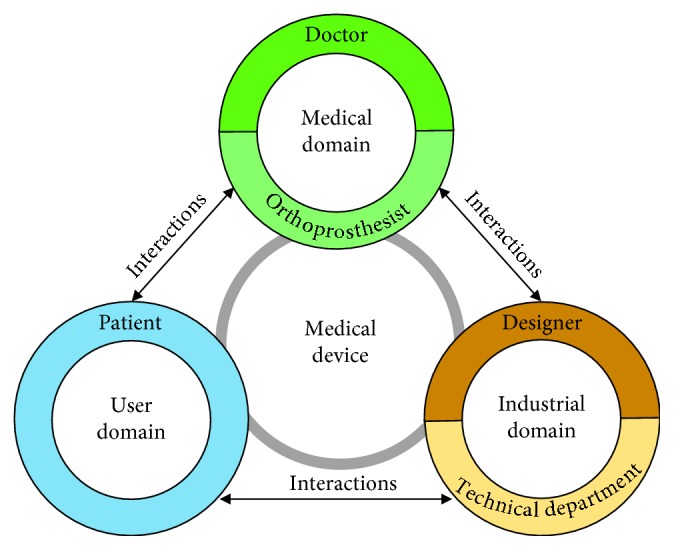
Orthosis development stakeholders.

Orthosis development should include several stakeholders from different domains, according to the complexity and requirements of the device. Based on this, the development of medical devices such as orthoses may consider stakeholders from three domains: *medical domain, industrial domain, and user domain* (Figure 1).

- Medical domain: there are two main actors with respect to the medical domain, the doctor and the orthoprosthesist. The doctor is responsible for the diagnosis and the medical design specifications of the orthosis. The orthoprosthesist, working with the other members of the design team, is responsible for the geometrical materialization of the support in the orthosis and adapting it to the patient's body.
- Industrial domain: there are two main actors with respect to the industrial domain, the mechanical designer and the technical department. The mechanical designer checks the specifications of the medical domain, and then, with the technical department, he develops the concept and the functional prototypes in the early design stages. Later, both the actors produce the orthosis.
- User domain: since an orthosis is in strict contact with the patient's body, patient feedback is key throughout the design process in order to include comfort and ergonomic criteria.

### 2.2. Orthosis Typology

Orthoses can be divided into different families according to the criteria and functions considered. By taking into account whether or not there is a mechanism to connect the several supports of the orthosis and which consequently displaces one with respect to the other, orthoses can be divided into two groups: orthoses with no mechanism (ONM) and orthoses with mechanism (OWM).

**Figure 2:**
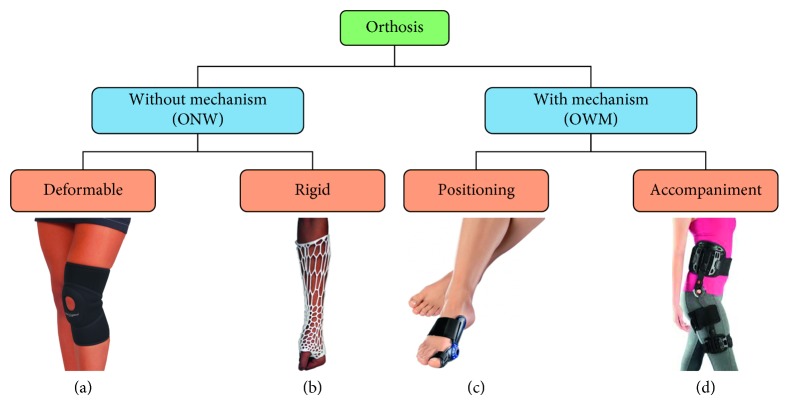
Orthosis families ((a) knee orthosis; (b) arm orthosis; (c) toe feet orthosis; (d) leg orthosis).

These groups can be subdivided still further. The ONM can be divided into rigid (e.g., plaster) and deformable (e.g., elastic knee brace), while the OWM can be divided into positioning (e.g., foot orthosis) and accompaniment (e.g., leg orthosis) (Figure 2).

Each type of OWM can be divided into two main components: supports and mechanism. Additionally, the supports can be divided into initial support (IS) and final support (FS). The body segment in contact with the supports is called the reference. The designation of initial reference (IR) and final reference (FR) is used according to the support that it is connected with [2]. Next, orthosis usage can be divided into different stages, called life situations, whereby their role can be precisely identified and understood.

### 2.3. Life Situations

**Figure 3:**
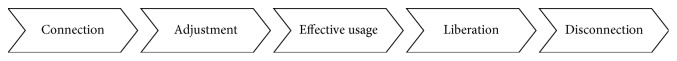
Orthosis usage life situations.

The notion of life situation involves breaking down the different moments during the use of a device [9–11]. Life situations correspond to the direct and indirect interactions between the user and the device when the orthosis is in use [11]. Based on this definition, the orthosis usage cycle can be divided into five distinct situations (Figure 3).

*Connection* corresponds to the positioning of the orthosis with respect to the body segment. This means that each support will be placed upon the reference. *Adjustment* corresponds to the phase where the final position of the supports will be defined and consequently linked with the medical prescription in order to correct the pathology. The third phase, *usage*, corresponds to the effective use of the orthosis. *Liberation* corresponds to the phase where adjustment, defined previously, is released. Finally, *disconnection* corresponds to the removal of the orthosis [11]. The particularity of this division lies in the fact that for an orthosis without a mechanism, there are no “adjustment” and “liberation” life situations.

The definition of the life situation of the orthosis marks the end of the task clarification stage and the beginning of the orthosis development.

### 2.4. Methodology

The proposed methodology is composed of six stages: *mechanism research; concept, displacement, and dimensional constraints; comfort adaptations; blocking definition; orthosis definition; and orthosis design qualification*.

**Figure 4:**
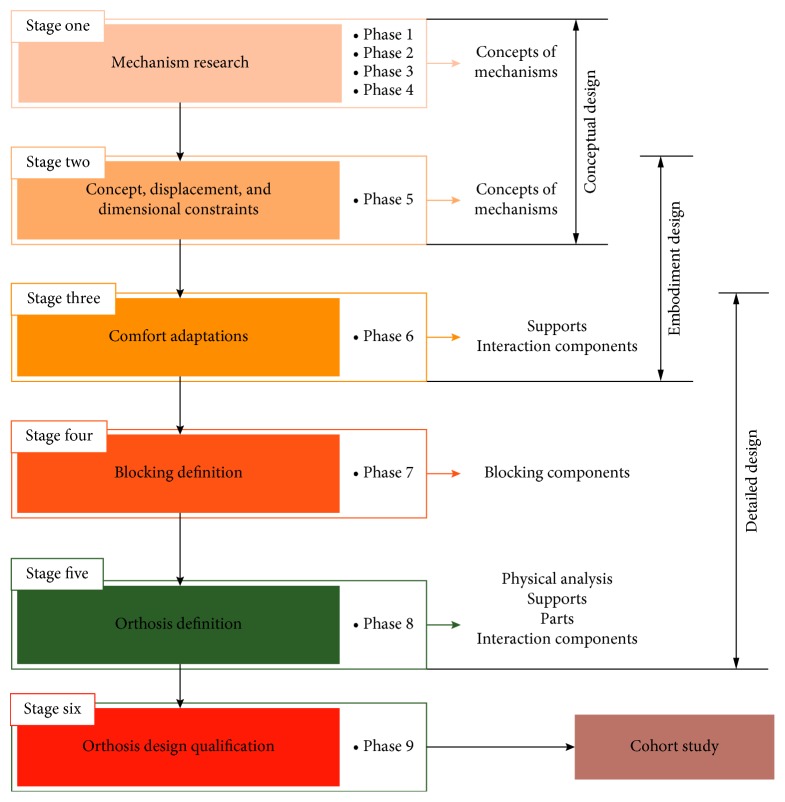
Orthosis design methodology.

These stages can be arranged according to the definition given by Pahl and Beitz (Figure 4) [7], however, unlike their proposition, the methodology proposed here considers a superposition of stages for a more dynamic design process.

The methodology will be explained in the next sections of this paper and the final overview presented at the end of the Materials and Methods section.

#### 2.4.1. Stage One: Mechanism Research

The aim of the first stage (Figure 4), which is part of the conceptual design phase, is to perform an exhaustive mechanism research in order to guarantee the desired mobility between the initial and the final orthosis support. This research takes into account the principles of the theory of mechanisms [11–13].

The first stage is divided into four phases where the designer defines the following:

- The number of solids included in the kinematics chain
- The kinematics between the supports
- The dependency between translations and rotations (when the rotation generates small translations)
- The usefulness of considering additional degrees of freedom in the kinematics chain to what is necessary in order to establish a nonessential DoF for the limb
- The advantage of having superabundant degrees of freedom

#### 2.4.2. Stage Two: Concept, Displacement, and Dimensional Constraints

The second stage (Figure 4) is between the end of the conceptual stage and the start of the embodiment design stage. The aim is to define

- displacement constraints, taking into account the medical prescription
- dimensional constraints, taking into account the patient's morphology
- general design constraints

#### 2.4.3. Stage Three: Comfort Adaptations

The third stage (Figure 4) comes between embodiment design and detailed design. At this stage, there are iterations between the designer and the patient in order to manage the comfort issues. The designer starts by deciding the type of orthosis (accompanying or fixed position).

During this stage, the mechanical designer analyzes the hyperstatism problems related to the type of orthosis. In the case of an orthosis, hyperstatism can cause local discomfort due to parasite efforts at the links between elements.

**Figure 5:**
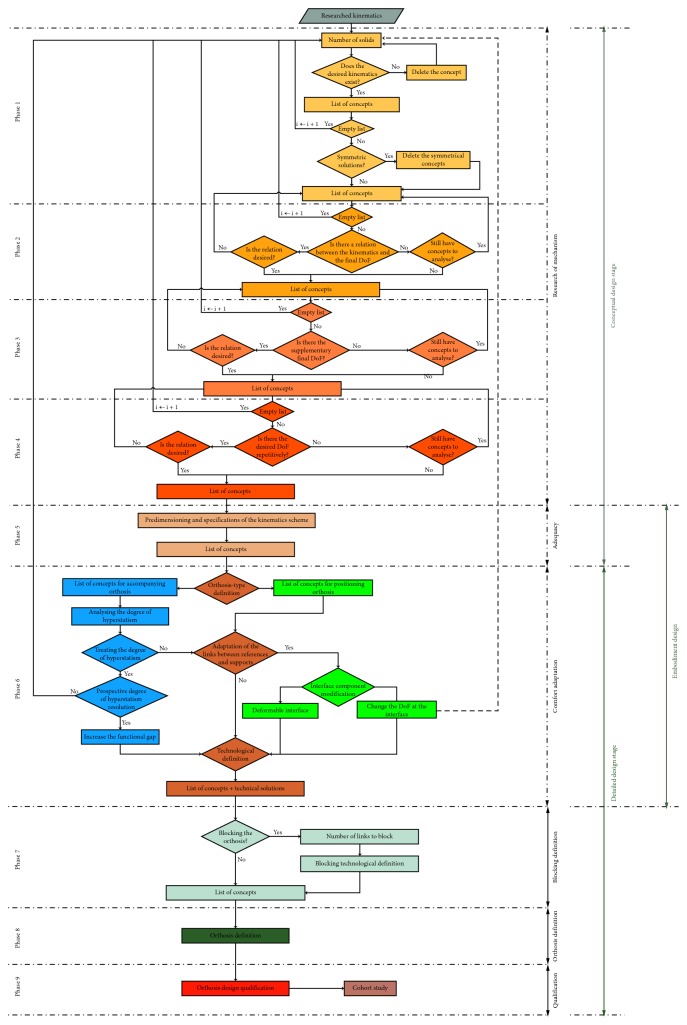
Orthosis methodology synoptic.

Both types of orthosis need to be analyzed independently, and for this reason, the proposed methodology considers a bifurcation at this stage (Figure 5).

**Figure 6:**
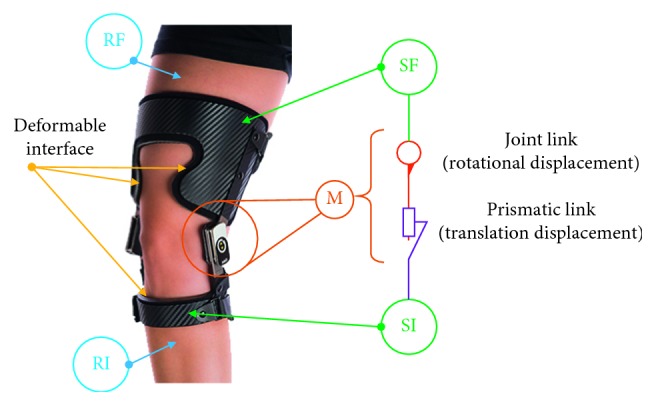
Accompaniment orthosis Odra (http://www.odra.ca).

*(1) Accompaniment Orthosis*. As the accompaniment orthosis moves with the limb, the hyperstatism may depend on the nature of the links and the solid chain size. For this reason, this stage starts by the hyperstatic analysis of the chain. To explain the particularities of this type of orthosis, Figure 6 shows an example of an accompaniment orthosis which allows an approximately natural movement of the leg (six degrees of freedom between tibia and femur).

The mechanical designer may choose to treat the degree of hyperstatism by changing the interface between the references and the supports or by modifying the functional gap and tolerances. If the mechanical designer chooses to change the interface between the references and the supports, he has two options: first, he can increase the number of links in order to unblock relevant DoF and second, he can introduce deformable links.

By modifying the functional gap, the mechanical designer is able to change locally the small displacements allowed by the links, which increases the mobility of the link and reduces the hyperstatism of the chain.

**Figure 7:**
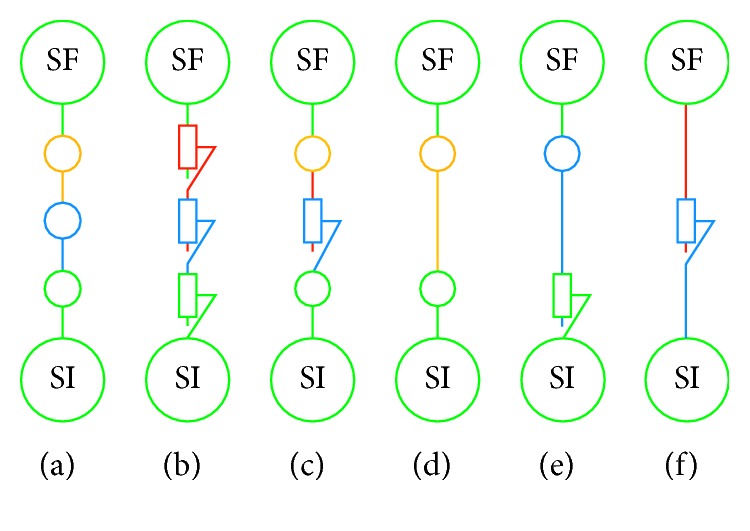
The mechanical chains obtained.

*(2) Positioning Orthosis*. The positioning orthosis respects a predefined position according to use. This type of orthosis blocks the kinematics of the limb, and then, the degree of hyperstatism is independent of the number of links in the chain (Figure 7).

In this case, the mechanical designer may treat the hyperstatism problems by introducing solids and links or introducing deformable links between the references and the supports.

#### 2.4.4. Stage Four: Blocking Definition

The fourth (Figure 4) stage corresponds to the detailed design phase. During this stage, the designer defines the way to suppress the degrees of freedom (DoF) of the mechanism, consequently blocking it, which locks the orthosis in its prescribed treatment position.

Based on this, at this stage, the designer defines

- the number of links that should be blocked
- how to block these links

#### 2.4.5. Stage Five: Orthosis Definition

In the fifth stage (Figure 4), which corresponds to the detailed design stage, the complete orthosis is defined. At this point, the designer needs to choose from the company's database or design all the components. After this, it is also necessary to ensure that all the components are assembled correctly.

#### 2.4.6. Stage Six: Orthosis Design Qualification

The last stage (Figure 4) corresponds to the orthosis design qualification, in which the designer evaluates the orthosis, through the user's inquiries. At this stage, possible changes can be made to future orthosis models but the orthosis can also be personalized according to the user's needs.

An extended synopsis is presented in Figure 5, to provide details of all the design decisions needed during the proposed methodology and to guide the mechanical designers in their choices.

This methodology was validated through a specific case study, proposed by Lagarrigue. The objective was to develop a postural brace to treat a postural disorder called camptocormia or bent spine syndrome, which affects elderly populations.

## 3. Results

**Figure 8:**
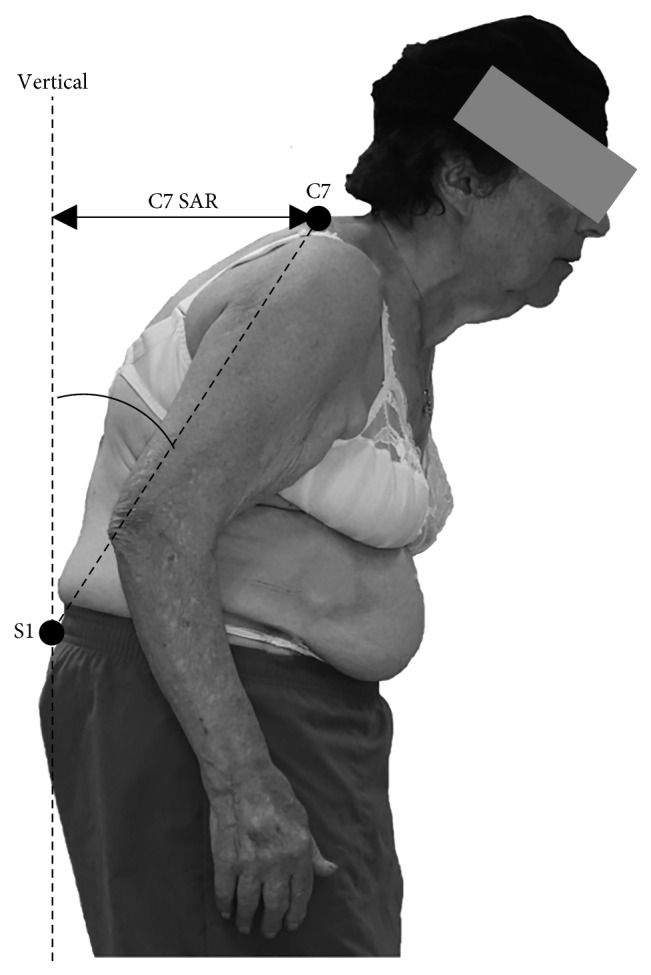
Camptocormia patient's typical posture.

The methodology was then applied to the development of a brace to treat a postural pathology named camptocormia. Also known as bent spine syndrome, it is characterized by the progressive anteroflexion of the trunk during walking and in the standing position (Figure 8) [14–18]. Contrary to other postural disorders, camptocormia is reversible, which means that the postural flexion is not permanent and patients are able to redress their posture by pushing the ties with the hand or standing against a wall [18–20].

Even if presently the etiologies of camptocormia are not completely understood by the medical community, and still exists several questions without answers, the treatment combining physiotherapy sessions with a brace has presented satisfying results [21–27]. Yet, as the camptocormia is a multicasual postural disease, these causalities were considered during the orthosis design process.

From the stakeholders' definition, the design team in accordance with the medical domain establishes the orthosis specification (Table 1). These specifications correspond to the starting point of the methodology.

From the specifications, it was observed that the orthosis should permit movement between the orthosis supports in order to promote the redress of the trunk.

According to the classification described above (Figure 2), this brace is considered as an orthosis with a mechanism which establishes a predefined position. The orthosis life situations can then be divided into connection, adjustment, effective usage, liberation, and disconnection (Figure 3).

The methodology was applied while considering the specifications established in Table 1. During the mechanism research, we considered that there can be three solids that make up the mechanism (articulation) between the initial and the final support.

**Figure 9:**
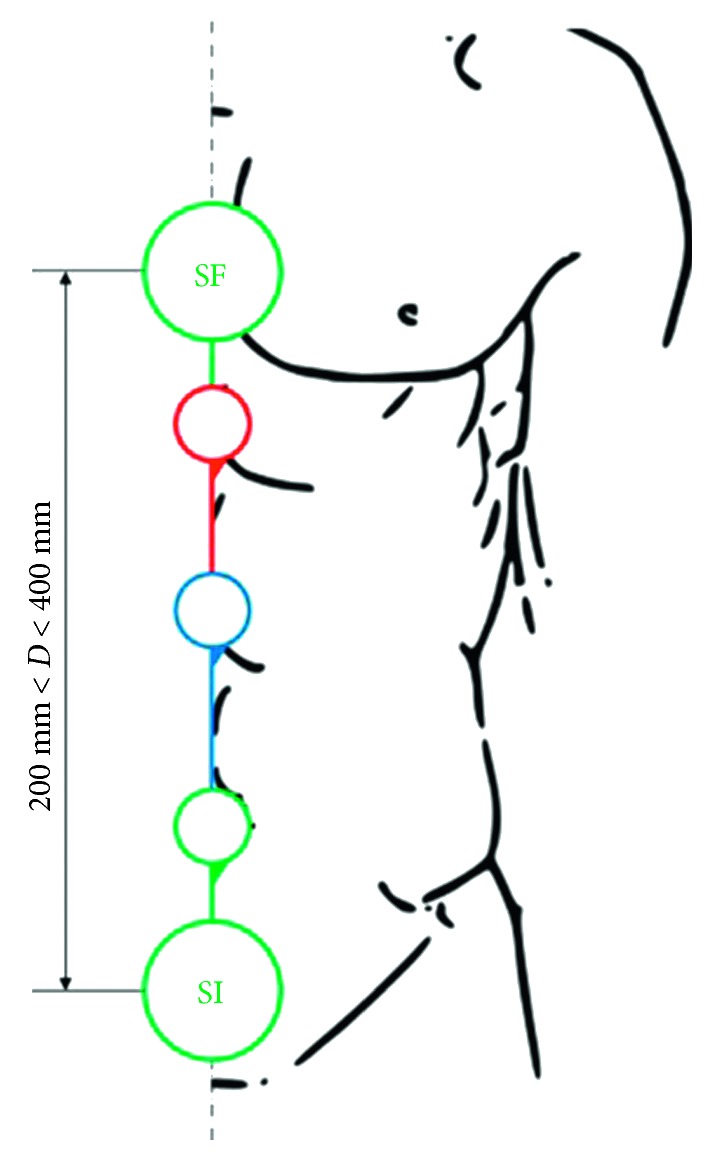
Orthosis mechanism chain (three joints) dimensioning.

In order to propose the optimal solution, an exhaustive mechanism database was used, as developed in Duarte's PhD thesis [28], which correlates the number of links and the number of solids with the desired kinematics of the chain. As a disclaimer, the authors of this work state that the purpose of this paper is not the explanation of the development of this database but its use along the proposed design methodology. So, by using this database, it is possible to identify and to choose the mechanism between the supports. During the first stage of the methodology, three solids and links were set. Consequently, the designer obtained a solution composed of three joints. The retained solutions were then dimensioned according to the users' needs, as defined in the specifications table (stage 2) (Figure 9).

At the third stage, *comfort adaptation*, the mechanical designer defined the type of orthosis, whether ONM or OWM. The brace treating camptocormia should maintain/restrain the body in a specific position which, according to the definition previously presented, represents a positioning OWM. During this stage, the mechanical designer defines the interface between the supports and the references in order to reduce adaptation problems due to morphological aspects and degrees of hyperstatism.

As the brace covers one of the body parts that frequently changes its morphology and the retained solution from the first stage represents an isostatic chain, in this specific case, the mechanical designer decided to introduce a deformable interface.

**Figure 10:**
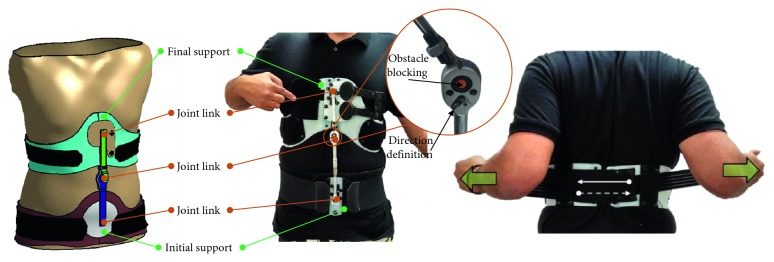
Camptocormia brace components.

Thus, during the *comfort adaptation* phase, the mechanical designer uses the materials database available in the enterprise to define the interface. At the same time, the interface option was laid on a neoprene fabric covering the orthosis support (Figure 10). Additionally, in order to improve comfort, and because the body frequently changes its morphology, adjustment strips were added to improve the connection between the supports and the body (Figure 10).

The following stage, *blocking definition*, defines the mechanism blocking system which locks the orthosis in the prescribed treatment position (trunk redressed). As at the anterior stage, the technical solution depends on the possibilities available to the enterprise. In this case, in accordance with the enterprise's blocking system database, the choice rests with an obstacle blocking system (Figure 10).

The next stage, *orthosis definition*, is based on the architectural design stage, which means assembling all the components of the orthosis. This stage is developed in strict contact with the user's domain since, depending on the geometry of the supports (a custom-made orthosis), the positioning of the mechanism may change. During this stage, numerical and experimental analyses are performed in order to evaluate the mechanical behavior of the orthosis.

The last stage, *orthosis design qualification*, corresponds to an evaluation of the orthosis by the user (user's domain). As previously described, during this stage, several questions were put to the user in order to evaluate the different components of the orthosis, for example, the supports, the mechanism, the blocking system, comfort, and ergonomics. The results of these inquiries not only enabled the designer first to make any necessary improvements but also to personalize the orthosis according to the patient's needs.

## 4. Conclusions

The development of medical devices is a demanding task, especially with respect to orthoses because of the way in which they are classified. In some cases, this results in deficient products that do not meet the patient's needs.

The primary aim of the present work was to develop an orthosis design methodology based on dividing up life situations and integrating the design constraints in different knowledge domains. This methodology was then applied to the development of a new orthosis, a brace to treat camptocormia.

Although several concepts emerged, in this study, only a representative group of specifications were considered, and for this reason, only one concept has been presented. The selected concept meets the main device specifications in terms of straightening the patient's posture.

This straightening was possible through the link chain proposed during the conceptual design stages and allows a vertical displacement of the chest. Concerning comfort, two aspects were considered. The first was the use of a neoprene layer between the rigid part of the supports and the body, and the second was the fact that with the proposed concept it is possible to tighten the supports to the body and consequently adjust the orthosis to the patient's morphology.

The blocking system of the proposed concept was specially taken into account in terms of ergonomics. Since the developed orthosis will be manipulated by elderly people and in some cases by patients with Parkinson's disease, the fact that their fine motor skills are often reduced must be considered.

## Figures and Tables

**Table 1:** Camptocormia brace specifications.

	No.	Functions	Criteria	Level
Mechanism	1	Admit a vertical adjustment	*Ty*	0 ≤ *Ty* ≤ 200 mm
	2	Be easily removable	Tasks	Tasks≤4
	3	Reduce the number of solids	Solids	Solids≤4
	4	Resist to the collapse of the trunk	Euler's force	Euler's force≤100 N
	5	Be lightweight	Mass	m≤300 g

Supports	6	Easy positioning	Steps	Steps≤5
		Reproducible positioning	Mass	m≤500 g
	7	Admit a morphological fit	Visual marks	Visual marks≥1
	8	Easy opening	Squashing	5%≤Squashing≤15%
	9	Easy positioning	Steps	Steps≤2

## Data Availability

As it is a design method, there are no medical data attached. We decided to use a case study related with camptocormia but in fact this methodology may be applied in any other orthosis mechanical designs. This means that the patient data analysis is not available.
